# Aggressive Vertebral Hemangioma: The Mystery of Spastic Legs Unveiled by a Purple Shoulder

**DOI:** 10.7759/cureus.21568

**Published:** 2022-01-24

**Authors:** Gurparvesh S Goraya, Sachi Singhal, Birinder S Paul, Gunchan Paul

**Affiliations:** 1 Neurology, Dayanand Medical College and Hospital, Ludhiana, IND; 2 Internal Medicine, Crozer-Chester Medical Center, Upland, USA; 3 Critical Care, Dayanand Medical College and Hospital, Ludhiana, IND

**Keywords:** vertebral hemangioma, magnetic resonance imaging, chronic dorsal myelopathy, cutaneous hemangioma, cavernous sinus hemangioma, aggressive vertebral hemangioma

## Abstract

Vertebral hemangiomas (VHs) are benign vascular tumors that develop from the endoderm of blood vessels, although their exact pathogenesis is poorly understood. Most hemangiomas are small, about a third are multiple in number, and a very small number of these hemangiomas cause symptoms. Even more rare are aggressive VHs, which comprise a small number of all VHs, and are associated with expansion and extraosseous extension into the paraspinal and epidural spaces. Management of aggressive VHs involve pre-op embolization, spinal surgery, and reconstruction. Pain management, physical rehabilitation, and close neurological follow-up are imperative to near-total recovery. Aggressive VHs are most commonly seen in the thoracic region but may rarely involve a large number of vertebrae. Cutaneous hemangiomas, when seen along with VHs, are often metameric.

We present a rare and challenging case of compressive myelopathy and a large cutaneous hemangioma or a "purple shoulder", found during an exam in a young male. He was found to have an extensive VH extending through 13 vertebral levels (C7 to D12), non-metameric to the cutaneous lesion. A thorough physical examination and evaluation along with prompt surgical treatment were the cornerstone of treatment and prevention of permanent neurological deficits.

## Introduction

Hemangiomas are benign, malformed vascular lesions that develop from the endoderm of blood vessels. Vertebral hemangioma (VH) is the most common benign tumor of the spine with an incidence of 10-12%. [1] A majority of these VHs are confined to the vertebral body. They are asymptomatic and either detected incidentally or not detected at all. A total of 0.9-1.2% of these benign vascular proliferative lesions present with symptoms such as back pain and neurological complaints. [2] Neurological symptoms, if any, may occur depending on the size and location of the tumor and the presence of bony erosion and spinal canal or neuroforaminal extension. Lesions displaying these features are called aggressive VHs and need prompt treatment. Assessing for radiculopathies and a methodical, detailed neurological exam are crucial for clinching the diagnosis. It is also imperative to differentiate them from metastatic lesions of the spine, Paget's disease, plasmacytomas, and other primary tumors of the spine.

## Case presentation

**Figure 1:**
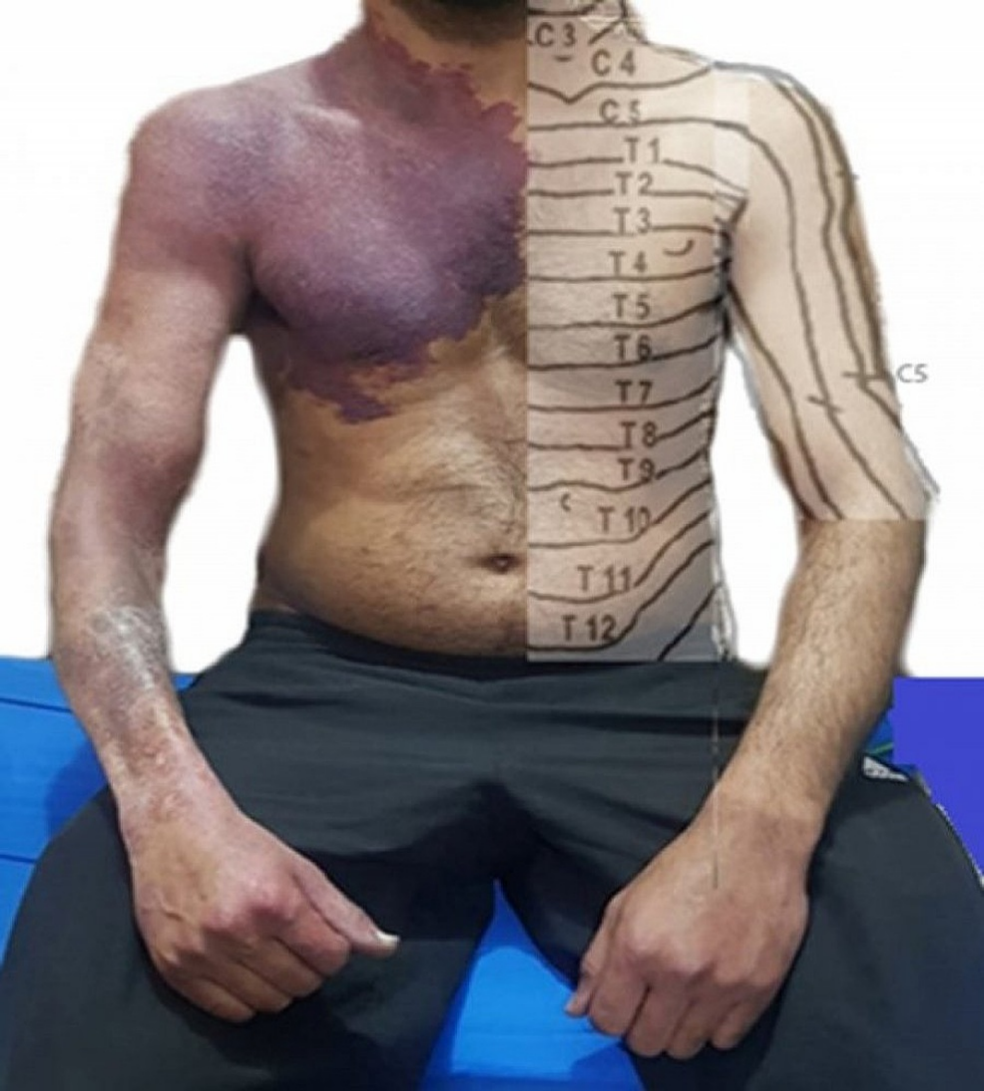
The large erythematous dusky raised red plaque with a bluish hue on the right upper trunk and arm involving C3 to C6, T1 to T5 dermatomes, and associated with distal forearm wasting. The anatomic depiction of corresponding dermatomes is done on the contralateral side for accurate picturization and comparison.

A 30-year-old male presented with complaints of walking difficulty and getting up from a squatting position. These symptoms began three months ago and were progressive. Along with progressive weakness, he complained of numbness that started in his feet and gradually ascended to his mid-thighs. On further questioning, he also reported urinary urgency but denied active or historical back pain. A thorough neurological examination was then undertaken, revealing normal mental status, intact cognition, and an unremarkable cranial nerve exam. Upper limb examination revealed distal forearm wasting on the right compared to the left. However, the motor exam was unremarkable, and power was symmetric and 5/5 on the Medical Research Council (MRC) muscle power scale bilaterally. The motor exam of the lower extremities was noted to be 4/5 on the MRC muscle power scale bilaterally. This decrease in power was associated with spasticity, brisk deep tendon reflexes, and bilateral extensor plantar responses. A sensory exam was then performed, revealing impaired sensations below the L1 spinal level. Other pertinent findings included a large erythematous dusky red plaque with a bluish hue on the right upper trunk and arm that the patient reported was present since childhood (Figure 1). The rest of his physical exams, including cardiopulmonary, GI, and genitourinary exams, were unremarkable. Based on these neurological findings, chronic thoracic myelopathy was diagnosed. 

**Figure 2:**
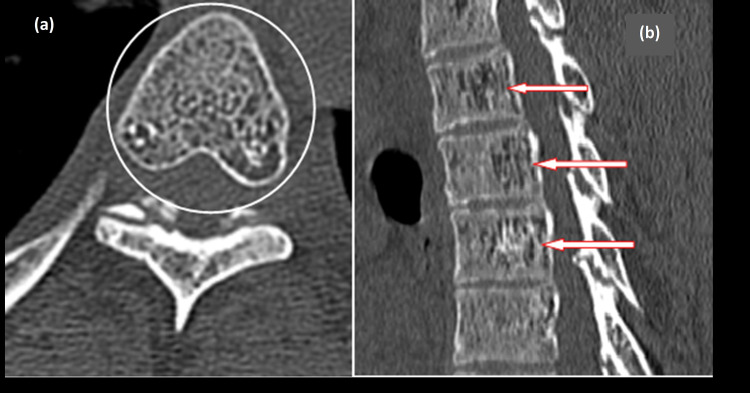
(a) Depicts an axial view of the CT scan showing classical polka dot appearance of the hemangioma, which is occupying most of the vertebral body and extending into both the pedicles and the lamina. (b) Shows a sagittal view of the same CT scan showing the classic "corduroy cloth" or "jail-bar" appearance of the vertebral bodies, highly indicative of a vertebral hemangioma

**Figure 3:**
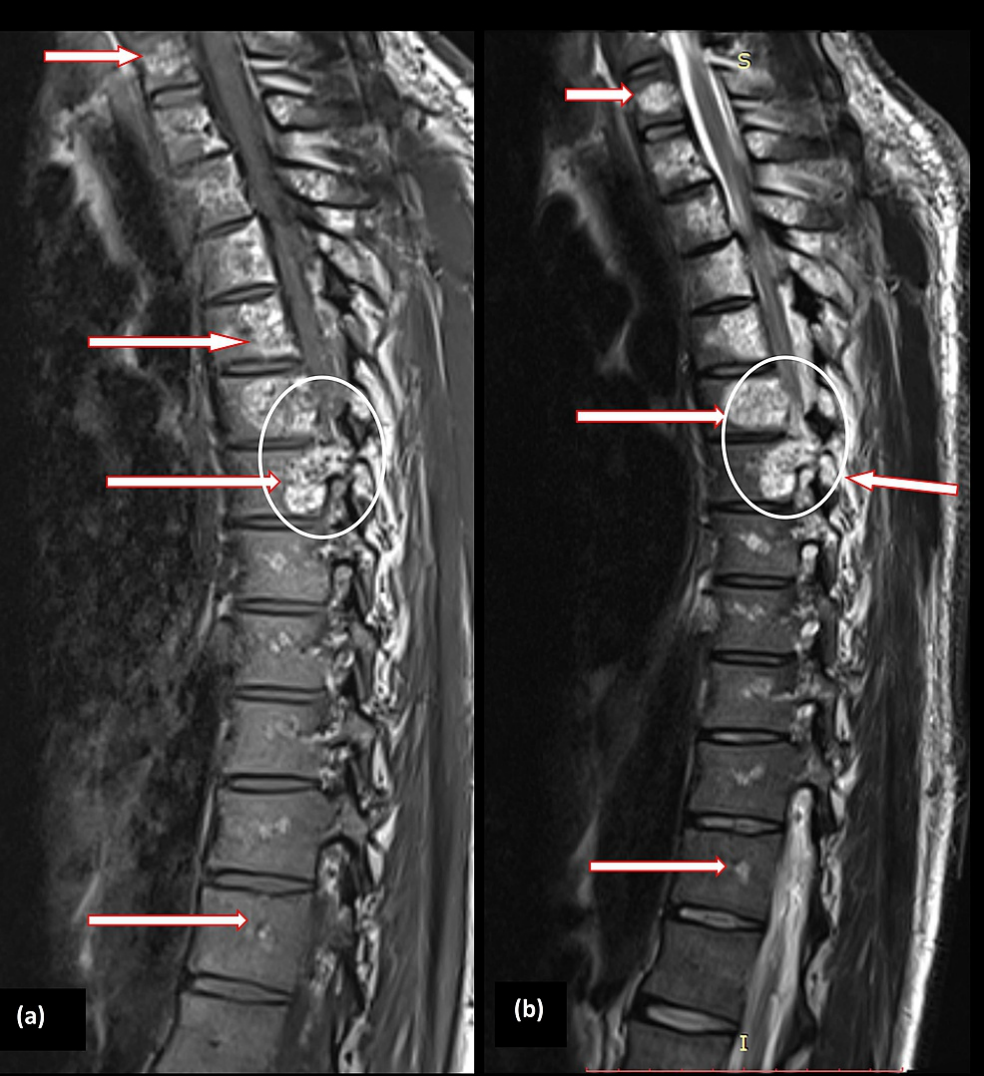
Sagittal views of MRI with contrast showing involvement of 13 vertebrae (marked with arrows in several places in both images (a) and (b)) from C7 to D12 with spinal cord compression (circled).

To further evaluate the etiology of this chronic dorsal myelopathy, a decision to obtain imaging was made. CT imaging showed coarse trabeculations, corduroy cloth appearance of the involved vertebra, consistent with the classic "jail-bar" sign highly suggestive of an intra-osseous hemangioma (Figure 2). To definitively assess this lesion, MRI with contrast was undertaken next, which revealed a patchy, heterogeneous epidural soft tissue mass extending into neural foramina, causing compression of thecal sac and cord. This tumor occupied most of the vertebral bodies between C7 and T10, involving pedicles, laminas, and the base of the transverse processes (Figure 3).

The patient reported worsening of symptoms in the subsequent days. In view of worsening spastic paraparesis with bladder and bowel involvement, the decision was made for him to undergo urgent decompression surgery. Cervical cord laminectomy from C7 to T4 with decompression of extradural hemangioma was done. The excised tissue was sent for the microscopic examination, which revealed large dilated and thin-walled vascular channels filled with blood and lined by flattened endothelial cells, along with intervening stroma of loose fibrotic tissue. This was consistent with our final diagnosis of cavernous hemangioma. The postoperative course of our patient was uneventful, and his spasticity improved, and his power gradually returned to a near-complete recovery. He was discharged after four weeks of extensive physiotherapy. He was consistently followed up in our specialized neurology clinic. On his most recent follow-up, three months after surgery, he was able to ambulate independently without any difficulty and reported a significant improvement in quality of life.

## Discussion

Hemangiomas are benign tumors of vascular origin, the pathogenesis of which is still poorly understood. Several postulated theories include growth factors and hormonal and mechanical influences primarily affecting the abnormal proliferation of endothelial cells. No genetic factors have been identified thus far [3]. Despite being the most common benign tumor of the spine, VHs are largely asymptomatic. Only less than 1% of adults report symptoms, which are most commonly pain (54%) or variable neurologic symptomatology (45%) [1]. These symptoms depend on age, degree of extension of the tumor, and the vascular component involved; and can range from radiculopathy to complete paralysis. VHs are deemed 'aggressive' when associated with vertebral expansion, significant extension, and other overt and alarming symptoms. The age of patients with VHs reported in the literature is variable, with an average age of 41.6 years. Our patient presented at the age of 30 years, falling close to the expected age range of presentation. Thoracic-cervical vertebrae are the most frequently involved site, and merely 13% of patients exhibited multi-level vertebral involvement. The most significant number of involved vertebrae in this study of 89 patients was nine [4]. In regards to aggressive VHs, a separate rare subset, 75% of these lesions occur exclusively in the thoracic spine [5]. VHs in the thoracic spinal region are more likely to be symptomatic due to that region's narrow canal dimensions. Hence, a more aggressive management plan is warranted before the onset of irreversible neurological deficits.

Our patient had an extensive VH involving about 13 vertebral bodies from C7 to T12. This is virtually unheard of in the existing literature. In addition to the extradural compression, a detailed interpretation of neuroimaging revealed cord ischemia, which might further explain intramedullary symptoms. Also, the presence of an extensive cutaneous hemangioma with associated wasting of the distal forearm corroborates the aggressive nature of this vascular lesion in our patient (Figure 1). This associated peripheral vascular malformation originates from the same embryonic segment, i.e., C5-T4, establishing a common dermatomal origin between these two lesions [6]. This signature lesion was the interesting clinical key to solving the neurological conundrum in our patient [7]. Vertebral vascular malformations in association with cutaneous vascular lesions are well described in the literature, but they primarily involve the same dermatomes as VHs [7-8]. A discrepancy between the cutaneous and spinal lesions has been very rarely reported in the literature. To the best of our knowledge, only one case of a 13-year-old female patient has been reported in the literature, with an aggressive VH and a non-metameric cutaneous hemangioma [9]. In our case, there was a relative mismatch between the presence of vertebral (C7-T10) and cutaneous hemangioma (C5,6-T2-4). Some common differentials for such lesions of the spine are sclerotic lesions from metastasis and Paget's disease. In particular, hemangiomas need to be differentiated from malignant/metastatic spine lesions as the management of the two widely varies. CT imaging is a good initial scan for evaluation. MRIs are generally considered the next step for a definitive assessment, diagnosis, and determination of extension, vascularity and compression from the VH in patients with abnormal findings [10]. In terms of management, most endovascular and non-operative treatments are available apart from operative resection. The highly vascular nature of the tumor associates it with high morbidity and mortality and a high risk of bleeding during surgery. However, pre-op embolization with surgical excision and reconstruction remains the mainstay of aggressive/compressive VHs [11]. Hence, more conservative strategies are preferred for small, asymptomatic hemangiomas or those without spinal canal invasion [12-13]. Our patient, with a highly unusual aggressive VH invading thirteen vertebrae, was treated with a surgical approach and managed in our specialized neurology clinic with close follow-ups, monitoring, and intensive physiotherapy and rehabilitation. 

## Conclusions

Vertebral hemangiomas are benign vascular tumors of the spine, with a prevalence of 10% in the general population. They are rarely, if ever, symptomatic. A thorough neurological exam is imperative in suspecting and diagnosing myelopathy and possibly aggressive VH. Occasionally, they are seen in a metameric fashion with cutaneous hemangiomas, and again, the importance of a comprehensive physical exam cannot be stressed enough. CT imaging is a preferable initial test to evaluate for VH and shows a plethora of characteristic findings. MRI can be considered to assess for extent and compression definitively. Treatment options are many and range from conservative management for asymptomatic VHs, to a multi-step surgical approach for aggressive lesions. Close follow-ups with a neurologist, a detailed neurological exam on each visit, and a focus on multi-modal therapies, including physiotherapy for strengthening, are imperative to make a full recovery.
